# Broadband and Incident-Angle-Modulation Near-Infrared Polarizers Based on Optically Anisotropic SnSe

**DOI:** 10.3390/nano13010134

**Published:** 2022-12-27

**Authors:** Zhengfeng Guo, Honggang Gu, Yali Yu, Zhongming Wei, Shiyuan Liu

**Affiliations:** 1 State Key Laboratory of Digital Manufacturing Equipment and Technology, Huazhong University of Science and Technology, Wuhan 430074, China; 2 Innovation Institute, Huazhong University of Science and Technology, Wuhan 430074, China; 3 Optics Valley Laboratory, Wuhan 430074, China; 4 State Key Laboratory of Superlattices and Microstructures, Institute of Semiconductors, Chinese Academy of Sciences, Beijing 100083, China; 5 Center of Materials Science and Optoelectronic Engineering, University of Chinese Academy of Sciences, Beijing 100049, China; 6 School of Optical and Electronic Information, Huazhong University of Science and Technology, Wuhan 430074, China

**Keywords:** near-infrared polarizers, optical anisotropy, SnSe, Mueller matrix spectroscopic ellipsometry

## Abstract

Optical anisotropy offers an extra degree of freedom to dynamically and reversibly regulate polarizing optical components, such as polarizers, without extra energy consumption and with high modulating efficiency. In this paper, we theoretically and numerically design broadband and incident-angle-modulation near-infrared polarizers, based on the SnSe, whose optical anisotropy is quantitatively evaluated by the complete dielectric tensor, complex refractive index tensor, and derived birefringence (~|Δ*n*|_max_ = 0.4) and dichroism (~|Δ*k*|_max_ = 0.4). The bandwidth of a broadband polarizer is 324 nm, from 1262 nm to 1586 nm, with an average extinction ratio above 23 dB. For the incident-angle-modulation near-infrared polarizer, the high incident angles dynamically and reversibly modulate its working wavelength with a maximum extinction ratio of 71 dB. Numerical simulations and theoretical calculations reveal that the considerable absorption for p light and continuously and relatively low absorption of s light lead to the broadband polarizer, while the incident-angle-modulation one mainly arises from the blue shift of corresponding wavelength of p light’s minimum reflectance. The proposed novel design of polarizers based on SnSe are likely to be mass-produced and integrated into an on-chip system, which opens up a new thought to design polarizing optical components by utilizing other low-symmetry materials.

## 1. Introduction

Owing to ultralow lattice thermal conductivity, tin (II) selenide, i.e., SnSe, crystals possess high thermoelectric figure of merit [1], which surpass many other thermoelectric materials [2,3,4]. Besides, SnSe is also a competitive candidate for solar cells [5,6], due to its suitable bandgap (about 0.86 eV for both theoretical [7,8] and experimental [1] studies), high absorption coefficient (at the level of 10^5^ cm^−1^ [9]), and relative abundance of composed elements [10]. More attractively, SnSe exhibits giant optical anisotropy, originating from its low-symmetry lattice structure analogous to the puckered structure of black phosphorus (BP) [11], and its symmetry of space group ($D_{2h}^{16}$ − *Pcnm* [12]) is even lower than that of BP ($D_{2h}^{18}$ − *Cmca* [13]). As reported, SnSe displays giant anisotropic absorption measured by angle-resolved polarized optical absorption spectroscopy, and the anisotropic ratio of absorption reaches up to 1.2 [8]. Optical anisotropy not only brings much richer physics such as birefringence [14,15] and dichroism [16,17], but also offers an extra degree of freedom to modulate its optical properties for polarization-related devices. However, most research on SnSe has been focused on the qualitative identification of optically anisotropic phenomena, and there remains a research gap and challenge to accurately acquire the complete dielectric tensor and the complex refractive index tensor to quantitatively evaluate the optical anisotropy of SnSe and then to rationally guide design of polarization-tunable optical components.

As optical components separating the polarization state, polarizers have been widely applied to display and imaging [18,19], optical interconnections and communications [20,21], and optical measurement [22,23]. In general, there are three main methods to produce polarizers. The first is to utilize some absorbing molecules (such as Iodine molecules [24] or liquid crystal molecules [18]), absorbing light along the preferred orientation, to realize the separation of the polarization state; the polarizers made by these absorbing molecules are frequently adopted to the field of display and imaging. However, on account of the instability chemicals, such polarizers may not be capable of working in the long term [25]. In addition, these polarizers are also subject to the problem of non-recyclability and environmental pollution [25]. Considering the demand for high levels of integration in optical interconnections and communications, the polarizers based on optical fibers [26,27] or optical gratings [28,29] are often highly integrated or directly integrated to platform of silicon on insulator (SOI) or lithium niobate on insulator (LNOI). However, such integrated polarizers based on optical fibers or optical gratings need to be elaborately designed, leading to expensive cost of design and fabrication. Although polarizers based on the simple-structure multilayer structure [30,31] is relatively low-cost, these polarizers seem difficult to become broadband, due to the limit of destructive interference. The last kind of polarizers are based on birefringent materials [32,33], with their applications in optical measurement, which separate the polarization state depending on the birefringence of materials or destructive interference in the multilayer structure. These simple-structure and low-cost polarizers generally do not require complex design. However, the polarizers based on birefringent materials are often fabricated with bulk single crystal, and there still remains a difficult and long way to implement miniaturization and integration of these polarizers as a consequence. Furthermore, once all the three kinds of polarizers have been produced, it is scarcely possible to dynamically and reversibly modulate their performance. Although polarizers made by liquid crystal can be tuned by external voltage to dynamically and reversibly regulate the polarization state [34,35], there still remain the tough problems of extra energy consumption and low modulating efficiency. Therefore, it is challenging to design low-cost and broadband polarizers with long-term stability and to efficiently and dynamically modulate their performance without extra energy consumption.

Herein, we quantitatively evaluate the optical anisotropy of SnSe by the Mueller matrix spectroscopic ellipsometry, and then rationally design the broadband and incident-angle-modulation near-infrared polarizers based on SnSe-SiO_2_-Si multilayer structure. Confirmed by the polarization-resolved reflectance spectra, the optical anisotropy of SnSe is then quantitatively characterized by the Mueller matrix spectroscopic ellipsometry to accurately acquire its complete dielectric tensor, complex refractive index tensor and derived birefringence and dichroism. The SnSe-SiO_2_-Si multilayer structure is constructed with its blue shift of corresponding wavelength of p light’s minimum reflectance and high reflectance of s light, suggesting significant potential to design high-performance and dynamically and reversibly modulated polarizers. By optimizing the thickness of SnSe and SiO_2_, the broadband and incident-angle-modulation near-infrared polarizers based on the SnSe-SiO_2_-Si multilayer structure are afterwards proposed by theoretical calculations and numerical simulations. Finally, the contour maps of electric field intensity and absorbed power of p and s light are numerically simulated to explore the mechanism for these broadband and incident-angle-modulation near-infrared polarizers.

## 2. Materials and Methods

### 2.1. Absorption Coefficient and Penetration Depth

The absorption coefficient *α_m_* (*m* = *b* or *c*) along *b*- or *c*-axis of SnSe is defined as [22]:

(1) $$\alpha_{m}=\frac{4\pi k_{m}}{\lambda },\;m=b\;\mathrm{or}\;c,$$

where *k_m_* (*m* = *b* or *c*) is the extinction coefficient along *b*- or *c*-axis of SnSe and *λ* is the wavelength with the unit of cm. 

The penetration depth *d*_p,*m*_ (*m* = *b* or *c*) along *b*- or *c*-axis of SnSe is the reciprocal of *α_m_* [22], i.e.,

(2) $$d_{p,\;m}=\frac{1}{\alpha_{m}},\;m=b\;\mathrm{or}\;c.$$

According to Beer’s law [22], the light intensity *I* after penetrating the sample, compared with the initial incident light intensity *I*_0_, is empirically calculated by:

(3) $$\frac{I}{I_{0}}=\exp(-\alpha d),$$

where *d* is the thickness of the sample. Therefore, the penetration depth *d*_p_ represents the depth that the light penetrates with its intensity $I_{d_{p}}$, reducing to about 37% of *I*_0_, combining the Equations (2) and (3):

(4) $$\frac{I_{d_{p}}}{I_{0}}=\exp(-\alpha d_{p})=1/e≅37\%.$$

### 2.2. Net Phase Shift of s Light

The net phase shift of s light $\phi_{\mathrm{net}}^{s}$ involves the propagation phase shift in the SnSe layer and reflective phases of SnSe’s upper and lower interfaces ($\phi_{\mathrm{upper}}^{s}$ and $\phi_{\mathrm{lower}}^{s}$), calculated by [36]:

(5) $$\phi_{\mathrm{net}}^{s}=2\phi_{\mathrm{prop}}^{s}+\phi_{\mathrm{lower}}^{s}-\phi_{\mathrm{upper}}^{s}=2\times \frac{2\pi d_{\mathrm{SnSe}}n_{y}\cos\theta_{\mathrm{air}-\mathrm{SnSe}}}{\lambda }+\phi_{{\mathrm{SnSe}-\mathrm{SiO}}_{2}-\mathrm{Si}}^{s}-\phi_{\mathrm{SnSe}-\mathrm{air}}^{s}$$

In Equation (5), the first term $\phi_{\mathrm{prop}}^{s}$ is the propagation phase shift of s light in the SnSe layer, and the *θ*_air-SnSe_ is the refractive angle at the interface between air and SnSe, which can be converted from Snell’s law [22]:

(6) $$N_{\mathrm{air}}\sin\theta =N_{c}\sin\theta_{\mathrm{air}-\mathrm{SnSe}},$$

where *N*_air_ (= 1) is the complex refractive index of air and *N_c_* is the SnSe *c*-axis’s complex refractive index.

The second term $\phi_{\mathrm{lower}}^{s}$ is the reflective phase for s light from the SnSe layer to the SiO_2_ layer, and then to Si substrate, which can be acquired by calculating the argument of amplitude reflection coefficient of s light $\phi_{{\mathrm{SnSe}-\mathrm{SiO}}_{2}-\mathrm{Si}}^{s}$ for the corresponding SnSe-SiO_2_-Si multilayers, utilizing the transfer matrix method [37], i.e.,

(7) $$r_{{\mathrm{SnSe}-\mathrm{SiO}}_{2}-\mathrm{Si}}^{s}=\left|r_{{\mathrm{SnSe}-\mathrm{SiO}}_{2}-\mathrm{Si}}^{s}\right|\phi_{{\mathrm{SnSe}-\mathrm{SiO}}_{2}-\mathrm{Si}}^{s}.$$

In Equation (7), $r_{{\mathrm{SnSe}-\mathrm{SiO}}_{2}-\mathrm{Si}}^{s}$ is the amplitude reflection coefficient of reflected s light for SnSe-SiO_2_-Si multilayers.

The third term $\phi_{\mathrm{upper}}^{s}$ is the reflective phase for s light from SnSe layer to air and equals to the argument of the reflected s light’s amplitude reflection coefficient from SnSe layer to air $\phi_{\mathrm{SnSe}-\mathrm{air}}^{s}$, i.e.,

(8) $$r_{\mathrm{SnSe}-\mathrm{air}}^{s}=\left|r_{\mathrm{SnSe}-\mathrm{air}}^{s}\right|\phi_{\mathrm{SnSe}-\mathrm{air}}^{s}.$$

According to Fresnel equations [22], the reflected s light’s amplitude reflection coefficient from SnSe layer to air $r_{\mathrm{SnSe}-\mathrm{air}}^{s}$ can be expressed as:

(9) $$r_{\mathrm{SnSe}-\mathrm{air}}^{s}=\frac{N_{y}\cos\theta_{\mathrm{air}-\mathrm{SnSe}}-N_{\mathrm{air}}\cos\theta }{N_{y}\cos\theta_{\mathrm{air}-\mathrm{SnSe}}+N_{\mathrm{air}}\cos\theta }.$$

### 2.3. Theoretical CSalculations by the 4 × 4 Matrix Method

The 4 × 4 transfer matrix **T** can be expressed in matrix form [22,38]:

(10) $$T=\left[\begin{array}{cccc} T_{11} & T_{12} & T_{13} & T_{14}\\ T_{21} & T_{22} & T_{23} & T_{24}\\ T_{31} & T_{32} & T_{33} & T_{34}\\ T_{41} & T_{42} & T_{43} & T_{44} \end{array}\right].$$

The reflectance of s and p light *R*_s_ and *R*_p_ of the SnSe-SiO_2_-Si multilayers can be thus calculated with the matrix elements of **T** [39]:

(11) $$\begin{array}{l} R_{s}=r_{\mathrm{ss}}^{2}+r_{\mathrm{sp}}^{2}\\ \;\;\;\;={\left(\frac{T_{21}T_{33}-T_{23}T_{31}}{T_{11}T_{33}-T_{13}T_{31}}\right)}^{2}+{\left(\frac{T_{11}T_{23}-T_{13}T_{21}}{T_{11}T_{33}-T_{13}T_{31}}\right)}^{2} \end{array}$$

(12) $$\begin{array}{l} R_{p}=r_{\mathrm{ps}}^{2}+r_{\mathrm{pp}}^{2}\\ \;\;\;\;={\left(\frac{T_{33}T_{41}-T_{31}T_{43}}{T_{11}T_{33}-T_{13}T_{31}}\right)}^{2}+{\left(\frac{T_{11}T_{43}-T_{13}T_{41}}{T_{11}T_{33}-T_{13}T_{31}}\right)}^{2} \end{array}$$

In Equations (11) and (12), *r*_ss_ represents the amplitude reflection coefficient of reflected s light, while *r*_sp_ is that of reflected p light induced by incident s light. Similarly, *r*_ps_ and *r*_pp_ respectively show the amplitude reflection coefficient of reflected s and p light with the incidence of p light. 

### 2.4. Numerical Simulations Using the Finite-Difference Time-Domain (FDTD) Method

The FDTD is a power way to simulate the reflectance and distribution of the electric field and absorbed power of a multilayer structure [40,41]. A very high mesh accuracy of 8 has been adopted to well match the theoretical calculation results. The incident plane wave is along the negative direction of the *z*-axis that is parallel to the vibration direction of the p light’s vertical component. Owing to the oblique incidence, the Bloch boundary conditions have been employed along *x* and *y* directions, which are respectively parallel to the vibration direction of the p light’s horizontal component and s light. The perfectly matched layer (PML) boundary condition has been utilized along the *z* direction.

## 3. Results and Discussion

### 3.1. Optical Anisotropy of SnSe

**Figure 1:**
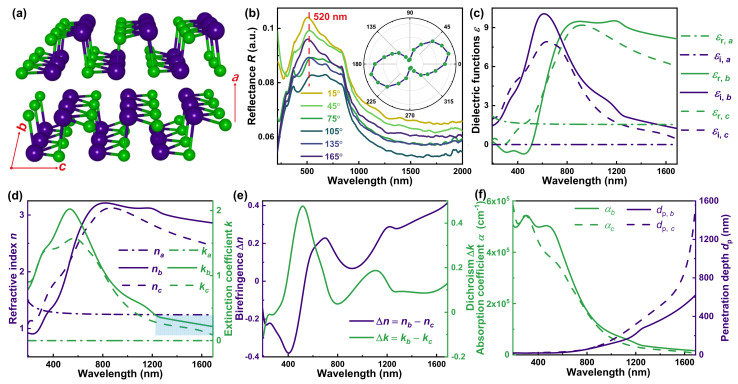
The optical anisotropy of SnSe. (**a**) The low-symmetry lattice structure of SnSe. (**b**) The polarization-resolved reflectance spectra with insert of the corresponding polar coordinate curve at peak wavelength of 520 nm. (**c**) The dielectric tensor, (**d**) the complex refractive index tensor, and (**e**) the derived birefringence Δ*n* and dichroism Δ*k* of *b*-*c* plane of SnSe. (**f**) The absorption coefficient *α* and the penetration depth *d*_p_ along the *b*- and *c*-axis, respectively, of SnSe.

Figure 1a illustrates the low-symmetry orthorhombic lattice structure of layered SnSe. Analogous to BP’s structure, the van der Waals (vdW) force connects each layer along the *a*-axis of SnSe [7], and the Sn and Se atoms alternately arrange and covalently constitute the zigzag and armchair structure along SnSe’s *b*- and *c*-axis, respectively [7,10]. This low-symmetry structure leads to the optical anisotropy of SnSe, identified by polarization-resolved reflectance spectra in Figure 1b. With a step of 30°, the reflectance spectra of SnSe vary distinctly with the polarization angle, suggesting the giant optical anisotropy. To clarify the relation between the reflectance and the polarization angle, the polar coordinate curve at peak wavelength of 520 nm is demonstrated in the insert of Figure 1b. The ratio of the maximum to the minimum is 1.26, indicating the SnSe’s giant optical anisotropy once again.

To quantitatively evaluate the optical anisotropy of SnSe, the Mueller matrix spectroscopic ellipsometry has been adopted to accurately acquire the complete dielectric tensor and the complex refractive index tensor of SnSe, demonstrated by Figure 1c,d, respectively. Details about ellipsometric analysis can be found in Appendix A. Since SnSe’s low-symmetry structure belongs to the orthorhombic crystal system, its dielectric tensor and complex refractive index tensor are diagonal and can be mutually converted by Equation (13) [38]:

(13) $$\left[\begin{array}{ccc} \varepsilon_{a} & 0 & 0\\ 0 & \varepsilon_{b} & 0\\ 0 & 0 & \varepsilon_{c} \end{array}\right]=\left[\begin{array}{ccc} N_{a}^{2} & 0 & 0\\ 0 & N_{b}^{2} & 0\\ 0 & 0 & N_{c}^{2} \end{array}\right]$$

where the subscript *a*, *b*, and *c* denote the dielectric functions *ε* and the complex refractive indices *N* along the *a*-, *b*-, and *c*-axis of SnSe, respectively. Furthermore, the dielectric function *ε* is composed of real part *ε_r_* and imaginary part *ε_i_*, i.e., *ε* = *ε_r_* − i*ε_i_*, while the refractive index *n* and the extinction coefficient *k* constitute the complex refractive index *N* (= *n* − i*k*). Therefore, the Equation (13) can be further expressed as:

(14) $$\left[\begin{array}{ccc} \varepsilon_{r,\;a}-i\varepsilon_{i,\;a} & 0 & 0\\ 0 & \varepsilon_{r,\;b}-i\varepsilon_{i,\;b} & 0\\ 0 & 0 & \varepsilon_{r,\;c}-i\varepsilon_{i,\;c} \end{array}\right]=\left[\begin{array}{ccc} {\left(n_{a}-ik_{a}\right)}^{2} & 0 & 0\\ 0 & {\left(n_{b}-ik_{b}\right)}^{2} & 0\\ 0 & 0 & {\left(n_{c}-ik_{c}\right)}^{2} \end{array}\right]$$

Since the vdW force between the layers is obviously different from the covalent interactions in the layers, a Cauchy model has been adopted to describe the dielectric function along the *a*-axis of SnSe [42], instead of the Tauc–Lorentz oscillators representing those along the *b*- and *c*-axis of SnSe [43] (See Appendix A for more details). Therefore, the dielectric function along the *a*-axis *ε_a_* and the complex refractive index *N_a_* show total differences from those along the *b*- and *c*-axis of SnSe in Figure 1c,d, respectively. Besides, the different arrangement of Sn and Se atoms along the *b*- and *c*-axis of SnSe possibly makes the corresponding dielectric functions and complex refractive indices discrepant in the peak positions and intensities, although they share almost the same peak shape. In brief, both dielectric functions and complex refractive indices show many differences along the direction of SnSe’s crystal axes, quantitatively demonstrating the giant optical anisotropy of SnSe. Shown in Figure 1e, we also calculate the birefringence Δ*n* (= *n_b_* − *n_c_*) and the dichroism Δ*k* (= *k_b_* − *k_c_*) to further quantitatively evaluate the optical anisotropy of SnSe. Both the birefringence Δ*n* and the dichroism Δ*k* display giant absolute value with their maximum about 0.4; the maximum absolute value of birefringence outperforms many other low-symmetry materials, such as BP (Δ*n*_BP_ = 0.15 [44]), ReS_2_ (Δ*n*_ReS2_ = 0.06 [45]), and α-MoO_3_ (Δ*n*_α-MoO_3__ = 0.11 [46]).

Moreover, the extinction coefficient *k* along the *b*- and *c*-axis of SnSe, i.e., *k_b_* and *k_c_*, descend gradually to zero above the wavelength of 1200 nm (marked area in Figure 1d), and this feature of weak absorption can be also observed from the absorption coefficient *α* and the penetration depth *d*_p_ along the *b*- and *c*-axis of SnSe in Figure 1f. Both *α_b_* and *α_c_* keep a high level of 10^5^ cm^±1^ in visible light region, analogous to the reported result [9], while they drop sharply to zero in the near-infrared region. The penetration depth *d*_p,*b*_ and *d*_p,*c*_ are also calculated to highlight the weak absorption feature, since they rapidly ascent in the near-infrared region. Besides, both the absorption coefficient *α* and the penetration depth *d*_p_ along the *b*- and *c*-axis of SnSe remain anisotropic, due to the anisotropic extinction coefficient *k* along the *b*- and *c*-axis of SnSe.

### 3.2. Design of Broadband and Incident-Angle-Modulation Near-Infrared Polarizers

**Figure 2:**
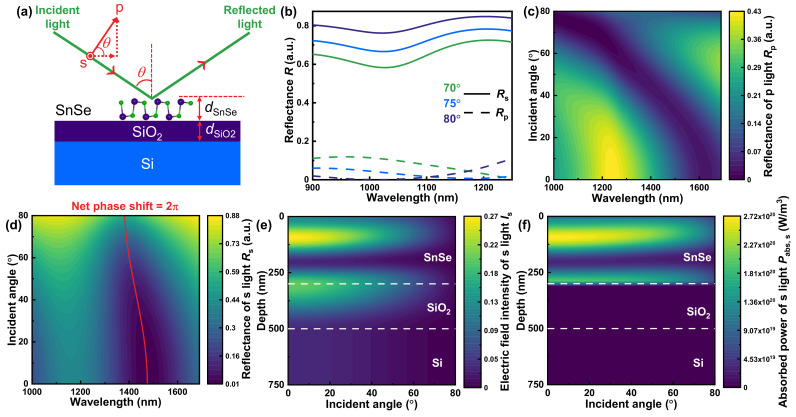
(**a**) SnSe-SiO_2_-Si multilayer structure with the incident angle *θ*. (**b**) The reflectance of s and p light at high incident angle of 70°, 75°, and 80°, where the thickness of SiO_2_ (*d*_SiO_2__) and the thickness of SnSe (*d*_SnSe_) are, respectively, 200 and 300 nm. The contour maps of reflectance of (**c**) p and (**d**) s light varied with the incident angle. The contour maps of s light’s (**e**) electric field intensity *I*_s_ and (**f**) absorbed power *P*_abs,s_ varied with the depth and the incident angle, selecting the maximum reflectance wavelength (1164 nm) at normal incidence.

**Figure A1:**
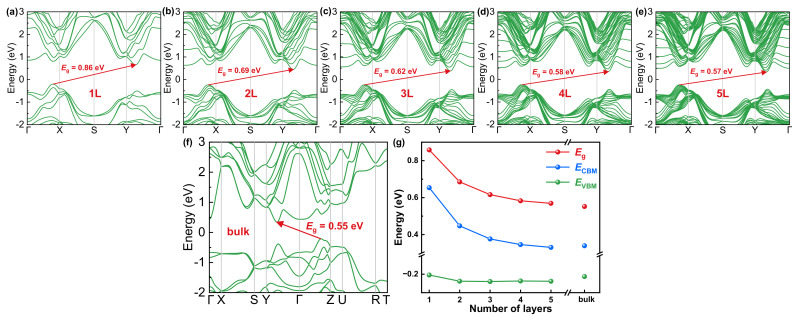
Band structure of 2D SnSe (**a**–**e**) from l layer to 5 layers (1 L–5 L) and (**f**) bulk SnSe calculated by first-principle, (**g**) bandgap energy *E*_g_, conduction band minimum (CBM) energy *E*_CBM_, and valence band maximum (VBM) energy *E*_VBM_ varied with the number of layers.

The weak absorption feature of materials, in most cases, gives rise to the destructive interference in their layer structure [47,48]. Hence, the polarization state can be separated by delaying the destructive interference of p or s light, utilizing optically anisotropic materials, such as BP [30,31]. Inspired by this idea, we constructed the SnSe-SiO_2_-Si multilayer structure (Figure 2a) as a reflective polarizer at incident angle *θ*. As a lossless dielectric material, SiO_2_ adds an extra degree of freedom to modulate the performance of the polarizer by altering its thickness (*d*_SiO_2__), other than that of SnSe (*d*_SnSe_). Additionally, the silicon (Si) substrate is selected due to its compatibility with integrated optoelectronics and on-chip system [28]. For convenience, the vibrational direction of s light and the horizontal component of p light keep parallel to the *c*- and *b*-axis of SnSe, respectively, all the time. The complex refractive index tensor adopted in the next theoretical calculations and numerical simulations is obtained from the SnSe single crystal sample, since we believe that several-hundred-nanometer-thickness SnSe’s properties may be more likely to that of bulk SnSe, which is proved by the thickness-dependent bandgap in Figure A1 of Appendix B.

As shown in Figure 2b, the reflectance of s light retains a high level (almost above 0.6) in the whole concerned wavelength range at high incident angle of 70°, 75°, and 80°. For p light, however, the reflectance is always below about 0.1, indicating that p light is probably able to be separated. More excitingly, the corresponding wavelength of minimum reflectance for p light is blue shift with increase of the incident angle, which offers significant potential to realize dynamical and reversible modulation.

Figure 2c illustrates the contour map of reflectance of p light varied with the incident angle to explain the blue shift phenomenon. Under a low incident angle (below about 20°), the horizontal component of p light, parallel to the *b*-axis of SnSe, plays a dominant role. Due to the fact that high refractive index of materials make their reflectance independent of the incident angle [49,50], the reflectance of p light remains unchanged within a low incident angle, due to the high refractive index along SnSe’s *b*-axis *n_b_* (≈3) in the near-infrared region. With the increase of higher incident angle, blue shift of corresponding wavelength of p light’s minimum reflectance comes up as a possible result of the refractive index along the *a*-axis *n_a_*, taking a more and more critical part in affecting the p light’s reflectance.

We also calculated the contour map of reflectance of s light, with the incident angle from 0° to 80° in Figure 2d, marking out the net phase shift of 2π. The net phase shift of 2π is the condition of destructive interference for the multilayer structure [36], and the corresponding wavelength of net phase shift of 2π perfectly matches with corresponding wavelength of minimum reflectance wavelength in Figure 2d. Therefore, for s light, destructive interference takes place in this SnSe-SiO_2_-Si multilayer structure. The high reflectance of s light in Figure 2b is probably a result of constructive interference. When the incident angle is below 60°, the s light’s reflectance is independent of incident angle, the same as p light’s circumstance under low incident angle, owing to the high refractive index along the *c*-axis [49,50]. However, reflectance of s light increases as the incident angle is beyond 60°. Figure 2e,f, respectively, demonstrate the contour maps of s light’s electric field intensity *I*_s_ and absorbed power *P*_abs,s_, varied with the depth and the incident angle at the representative constructive interference wavelength (1164 nm) to interpret the increase of s light’s reflectance. In Figure 2e, the electric field intensity *I*_s_ of SiO_2_ and SnSe maintain a consistently high level at low incident angle, while they decrease with the high incident angle. As to the absorbed power *P*_abs,s_, the absorption takes place almost totally in the SnSe layer, since SiO_2_ is a lossless dielectric material. Furthermore, the absorbed power *P*_abs,s_ in the SnSe layer shares the same trend as the electric field intensity *I*_s_, since *P*_abs,s_ is proportional to *I*_s_ in Equation (15) [51,52]:

(15) $$P_{\mathrm{abs},s}=\frac{1}{2}\omega \varepsilon_{0}I_{s}k_{c}$$

where *ω* is the angular frequency, expressed as *ω* = 2π*c*/*λ* (*c*: light speed in vacuum), and *ε*_0_ is the dielectric constant in vacuum. *k_c_* is the extinction coefficient along SnSe’s *c*-axis at the wavelength of 1164 nm, which can be regarded as a constant. Therefore, the absorption of the SnSe layer reduces with the increase of high incident angle, which leads to the high reflectance of s light.

**Figure 3:**
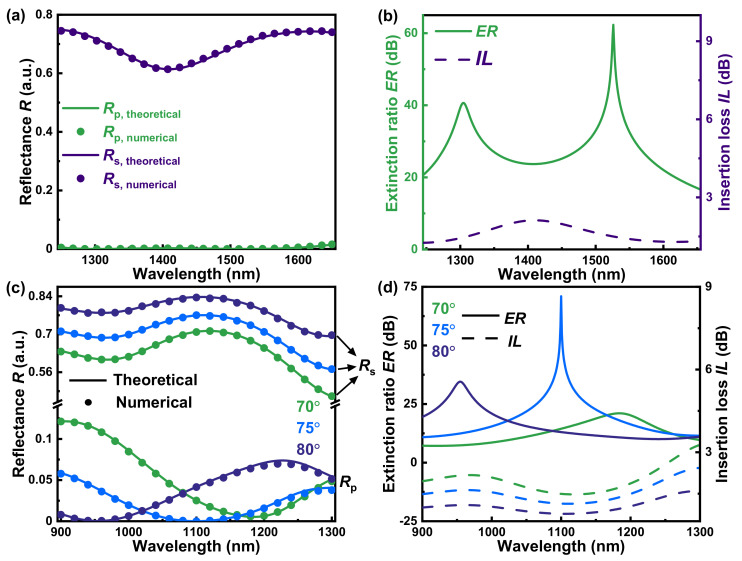
The broadband near-infrared polarizer based on SnSe-SiO_2_-Si multilayer structure with SnSe’s thickness of 400 nm and SiO_2_’s thickness of 675 nm: (**a**) the theoretical and numerical reflectance of p and s light at the incident angle of 75° and (**b**) the corresponding theoretical extinction ratio *ER* and insertion loss *IL*. The incident-angle-modulation near-infrared polarizer based on SnSe-SiO_2_-Si multilayer structure with SnSe’s thickness of 315 nm and the same SiO_2_’s thickness (*d*_SiO_2__ = 675 nm): (**c**) the theoretical and numerical reflectance of p and s light at the incident angle of 70°, 75°, and 80° and (**d**) the corresponding theoretical extinction ratio *ER* and insertion loss *IL* at the same incident angle.

By optimizing the thickness of SnSe and SiO_2_ (details in Appendix C), the broadband and incident-angle-modulation near-infrared polarizers based on optically anisotropic SnSe are acquired in Figure 3. For the broadband near-infrared polarizer based on SnSe (*d*_SnSe_ = 400 nm)-SiO_2_ (*d*_SiO_2__ = 675 nm)-Si multilayer structure, its reflectance of s light is above 0.6 at the incident angle of 75° within the whole concerned wavelength region in Figure 3a, which corresponds to the insertion loss *IL* below 2.1 dB. Herein, the *IL* of s light is defined as:

(16) $$IL=10\log_{10}\frac{1}{R_{s}}$$

More importantly, the p light’s reflectance *R*_p_ approaches zero in the almost concerned wavelength region in Figure 3a, with bandwidth of 324 nm for *R*_p_ < 0.003 (also shown in Table 1). Therefore, the corresponding extinction ratio *ER* is over 23 dB from the wavelength of 1262 nm to 1586 nm with the maximum extinction ratio *ER*_max_ of 62 dB in Figure 3b and Table 1. In this paper, the mentioned *ER* can be defined as:

(17) $$ER=10\log_{10}\frac{R_{s}}{R_{p}}$$

Compared to other near-infrared polarizers in Table 1, the proposed broadband polarizer possesses competitive *ER* and *IL*, as well as the longest bandwidth. Moreover, the numerical simulations on the reflectance of p and s perfectly match the theoretical results calculated by the 4 × 4 matrix method [22,38], which confirms the validity and reliability of this broadband near-infrared polarizer.

With regard to the incident-angle-modulation near-infrared polarizer based on SnSe-SiO_2_-Si multilayer structure, the optimized thickness of SnSe and SiO_2_ are, respectively, 315 nm and the same as that of the broadband near-infrared polarizer (*d*_SiO_2__ = 675 nm). It can be found in Figure 3c that the s light’s reflectance *R*_s_ keeps at a high level within all incident angles. For p light, the corresponding wavelengths of p light’s minimum reflectance are blue shift with the increase of the incident angle, which means that the incident angle is capable to dynamically and reversibly tune the working wavelength. The numerical simulations confirm this incident-angle-modulation polarizer as well in Figure 3c. In Figure 3d and Table 1, the corresponding wavelengths of p light’s minimum reflectance are, respectively, 1184, 1100, and 955 nm at the incident angle of 70°, 75°, and 80°, corresponding to the *ER*_max_ of 21, 71, and 35 dB and the *IL* of 1.7, 1.1, and 1.1 dB. Such performances of incident-angle-modulation polarizer are superior to most of the high-performance near-infrared polarizers in Table 1.

### 3.3. Mechanism for Broadband and Incident-Angle-Modulation Near-Infrared Polarizers

**Figure 4:**
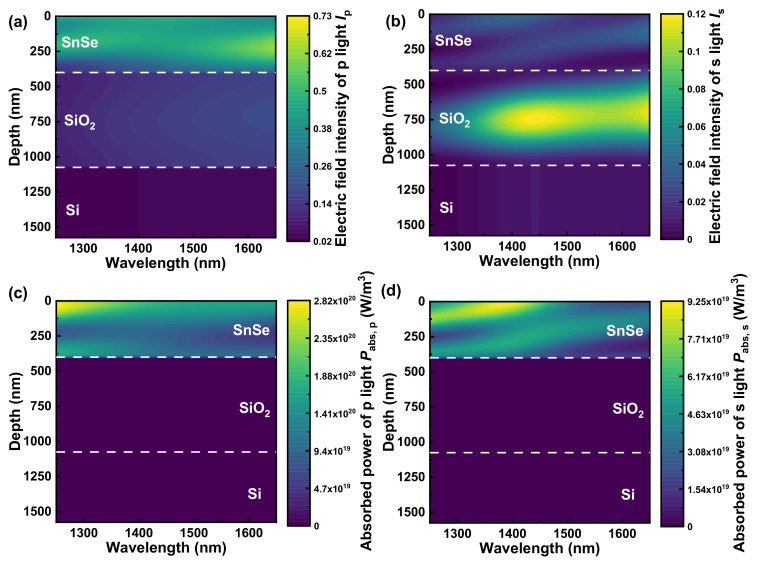
The mechanism for the near-infrared broadband polarizer based on SnSe (*d*_SnSe_ = 400 nm)-SiO_2_ (*d*_SiO_2__ = 675 nm)-Si multilayer structure. The contour maps of the electric field intensity of (**a**) p and (**b**) s light (*I*_p_ and *I*_s_), and the absorbed power of (**c**) p and (**d**) s light (*P*_abs,p_ and *P*_abs,s_) at the incident angle of 75°.

As illustrated in Figure 4, we have numerically simulated the contour maps of electric field intensity and absorbed power of p and s light (*I*_p_ and *I*_s_; *P*_abs,p_ and *P*_abs,s_) to explore the mechanism for the broadband near-infrared polarizer at first. The electric field intensity in the SnSe layer dominates the *I*_p_ in Figure 4a, while SiO_2_’s electric field intensity is dominated in *I*_s_ of Figure 4b. However, SiO_2_ is a lossless dielectric material, as previously mentioned. As a consequence, there is no *P*_abs,s_ in SiO_2_ layer of Figure 4d. Furthermore, the *I*_p_ in the SnSe layer maintains a relatively high level within the whole concerned wavelength region in Figure 4a. Eventually, the *P*_abs,p_ in the SnSe layer nearly keeps a high level of 1 × 10^20^ W/m^3^ through the entire concerned region in Figure 4c, contributing to the *R*_p_ approaching zero over broadband in Figure 3a. For s light, however, SnSe’s *I*_s_ in Figure 4b is nearly zero, and thus the *P*_abs,s_ of SnSe layer is approximately one order of magnitude lower than *P*_abs,p_ in the SnSe layer within the whole wavelength range. Consequentially, the s light’s reflectance *R*_s_ stays at a high level for the entire concerned range in Figure 3a, generating Figure 3b’s high-performance broadband near-infrared polarizer with comparatively low *IL* and high *ER*.

**Figure 5:**
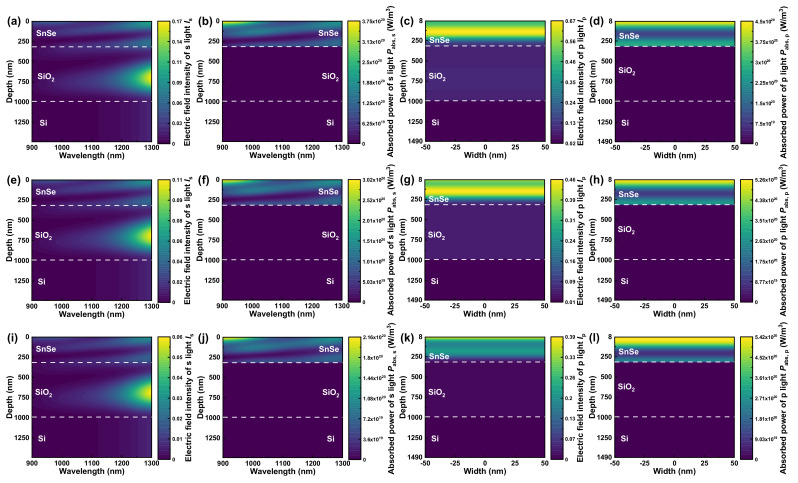
The mechanism for incident-angle-modulation near-infrared polarizer based on SnSe (*d*_SnSe_ = 315 nm)-SiO_2_ (*d*_SiO_2__ = 675 nm)-Si multilayer structure. The contour maps of electric field intensity of (**a**,**e**,**i**) s and (**c**,**g**,**k**) p light (*I*_s_ and *I*_p_) and absorbed power of (**b**,**f**,**j**) s and (**d**,**h**,**l**) p light (*P*_abs,s_ and *P*_abs,p_) at the incident angle of 70°/75°/80°, respectively, and *I*_p_ and *P*_abs,p_ is at the corresponding *ER*_max_ wavelength.

To investigate the mechanism for incident-angle-modulation near-infrared polarizer, the contour maps of electric field intensity and absorbed power of s and p light (*I*_s_ and *I*_p_; *P*_abs,s_ and *P*_abs,p_) with high incident angle from 70° to 80° have been numerically simulated in Figure 5. Despite the fact that *I*_s_ in longer wavelength is much higher than that in shorter wavelength in Figure 5a,e,i, *P*_abs,s_ displays nearly homogeneous distribution through the entire wavelength range. According to Equation (15), the *k_c_* in Figure 1d shows a declining trend during the near-infrared region, which leads to homogeneously distributed *P*_abs,s_ in almost concerned wavelength region. In addition, the average SnSe’s *P*_abs,s_ at specific incident angle (70°/75°/80°) in Figure 5b,f,j is about 2−4 times smaller than corresponding *P*_abs,p_ of Figure 5d,h,l in Table 2. According to the analysis above, *R*_s_ in Figure 3c is continuously and relatively high through the entire concerned wavelength range.

As regards p light, the average *I*_p_ in the SnSe layer decreases with increase of high incident angle from 70° to 80° at the *ER*_max_ wavelength in Figure 5c,g,k and Table 2. However, there seems to be no consistent trend in *P*_abs,p_, and *P*_abs,p_ sustains fairly high value at the *ER*_max_ wavelength throughout all the high incident angle in Figure 5d,h,l and Table 2. Herein, the *I*_p_ is constituted by quadratic sum of horizontal and vertical electric field component *E_x_* and *E_z_*, i.e., [56]

(18) $$I_{p}={\left|E_{x}\right|}^{2}+{\left|E_{z}\right|}^{2}.$$

Since the extinction coefficient along the *a*-axis (parallel to vibration direction of *E_z_*) *k_a_* is zero in Figure 1d, no absorption takes place along the vibration direction of *E_z_* accordingly. Thus, the *P*_abs,p_ can be represented as [51,52]:

(19) $$P_{\mathrm{abs},p}=\frac{1}{2}\omega \varepsilon_{0}I_{x}k_{b}.$$

**Figure A6:**
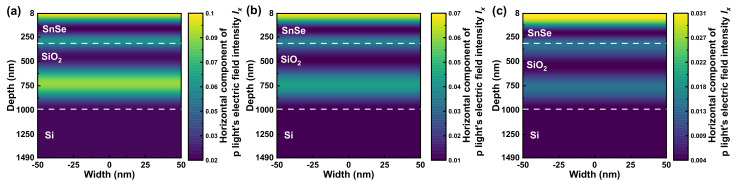
The contour maps of horizontal component of p light’s electric field intensity *I*_x_ in SnSe (*d*_SnSe_ = 315 nm)-SiO_2_ (*d*_SiO_2__ = 675 nm)-Si multilayer structure at the incident angle of (**a**) 70°, (**b**) 75°, and (**c**) 80° at the corresponding *ER*_max_ wavelength for incident-angle-modulation near-infrared polarizer.

In Equation (19), *I_x_* is the horizontal component of p light’s electric field intensity with the relation of *I_x_* = *|E_x_*|^2^. In Figure A6 of Appendix D, SnSe’s *I_x_* at *ER*_max_ wavelength also declines with the increase of high incident angle from 70° to 80°. However, the blue shift of *ER*_max_ wavelength makes the corresponding *k_b_* increase with the high incident angle since *k_b_* shares the same declining trend as *k_c_* in the near-infrared region of Figure 1d. Consequently, *P*_abs,p_ in the SnSe layer holds a considerable value throughout all high incident angle in Figure 5d,h,l and Table 2, producing the lowest *R*_p_ and corresponding *ER*_max_.

## 4. Conclusions

In summary, we have theoretically and numerically designed broadband and incident-angle-modulation near-infrared polarizers based on optically anisotropic SnSe. The giant optical anisotropy of SnSe is quantitatively revealed by the complete dielectric tensor, complex refractive index tensor, and derived birefringence (~|Δ*n*|_max_ = 0.4) and dichroism (~|Δ*k*|_max_ = 0.4), determined by the Mueller matrix spectroscopic ellipsometry. We find the weak absorption feature of SnSe in near-infrared region and the blue shift of the corresponding wavelengths of p light’s minimum reflectance in SnSe-SiO_2_-Si multilayer structure. Moreover, s light’s high reflectance originates from the constructive interference and reduction of absorption at high incident angle. All these features make the SnSe based multilayer structure become broadband, and incident-angle-modulation near-infrared polarizers with a bandwidth of 324 nm from the wavelength of 1262 nm to 1586 nm, and an average extinction ratio above 23 dB and dynamically and reversibly modulating by high incident angle with a maximum extinction ratio of 71 dB, respectively. The broadband polarizer originates from the results that p light’s absorption is one order of magnitude higher than that of s light throughout the entire concerned wavelength range. The numerical simulations reveal that the continuously and relatively low absorption of s light and the considerable absorption for p light at the high incident angle regulated absorption wavelength lead to high-performance and incident-angle-modulation polarizer in the near-infrared region. The SnSe-SiO_2_-Si multilayer structure may be easily fabricated by vacuum evaporation or magnetron sputtering, compared with manufacture process of the optical fibers or optical gratings structures. Such easily fabricated broadband and incident-angle-modulation near-infrared polarizers based on multilayer structure are likely to be mass-produced and can be used in integrated optics in practice. Our research opens up new thought to design other polarizing optical components by utilizing other low-symmetry materials.

## Figures and Tables

**Table 1:** Comparation of performances of near-infrared polarizers.

Polarizers	Extinction Ratio *ER* [dB]	Insertion Loss *IL* [dB]	Working Wavelength (Range) [nm]	References
Graphene fiber	~19 ± 2.5	~5	1530−1630 (100)	[26]
Graphene microfiber	31	-	1550	[53]
Subwavelength grating on SOI platform	>35	<0.6	1260−1390 (130) & 1520−1600 (80)	[28]
Subwavelength gratings on LNOI platform	>30	<3.1	1550	[29]
Hybrid plasmonic grating on LNOI platform	20	<2.3	1470−1700 (230)	[54]
Long-period grating on LNOI waveguide	20	<2	1430−1700 (270)	[55]
Broadband polarizer based on optically anisotropic SnSe	>23 (Maximum: 62)	<2.1 (Average: 1.7)	1262−1586 (324)	This work
Polarizer dynamically and reversibly modulated by high incident angle	21 & 71 & 35	1.7 & 1.1 & 1.1	1184 & 1100 & 955	This work

**Table 2:** The average SnSe’s *I*_p_ and *P*_abs,p_, with their ratio to *I*_s_ and *P*_abs,s_, respectively, at high incident angle and *ER*_max_ wavelength.

Incident Angle [°]	*ER*_max_ Wavelength [nm]	*I*_p_ [a. u.]	*I*_p_/*I*_s_	*P*_abs,p_/10^20^ [W/m^3^]	*P*_abs,p_/*P*_abs,s_
70	1184	0.45	15	2.29	2.75
75	1100	0.32	32	2.57	4.02
80	955	0.18	36	2.83	4.22

**Figure A2:**
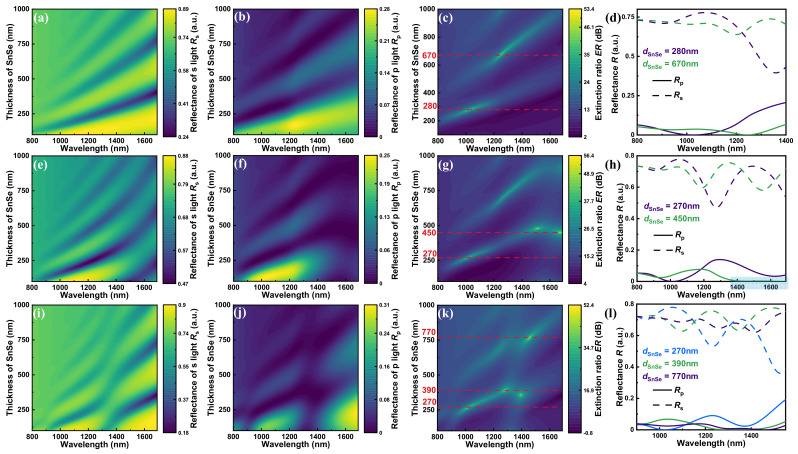
The contour maps of s and p light’s reflectance *R*_s_ and *R*_p_ and extinction ratio *ER* varied with the thicknesses of SnSe, selecting the the representative thicknesses of SnSe layer corresponding to the maximum extinction ratio *ER*_max_ to show their *R*_s_ and *R*_p_, respectively corresponding to the SiO_2_’s thickness of (**a**−**d**) 250, (**e**−**h**) 750, and (**i**−**l**) 1250 nm.

**Figure A3:**
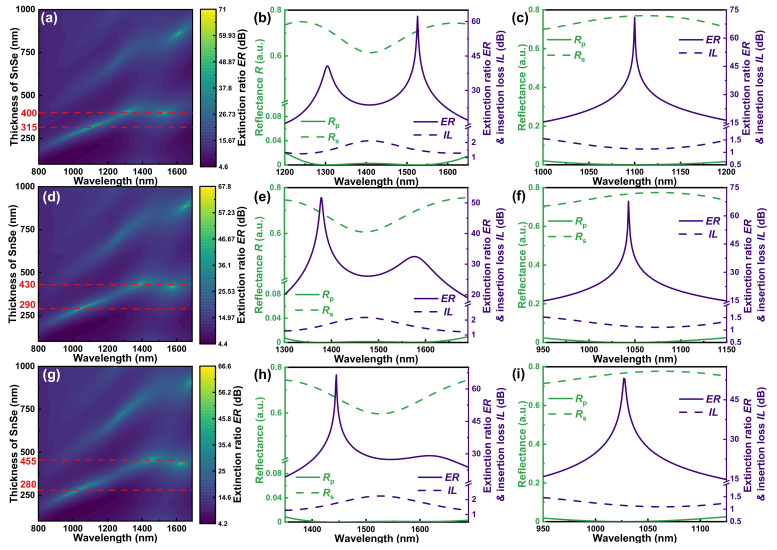
The contour maps of extinction ratio spectrum varied with the thickness of SnSe for the SiO_2_’s thickness of (**a**) 675, (**d**) 700, and (**g**) 725 nm, and the two representative thicknesses of SnSe layer (*d*_SnSe_ = (**b**) 400 and (**c**) 315 nm for SiO_2_’s thickness of 675 nm, (**e**) 430 and (**f**) 290 nm for that of 700 nm, and (**h**) 455 and (**i**) 280 nm for that of 725 nm) corresponding to the maximum value of extinction ratio *ER*_max_.

**Figure A4:**
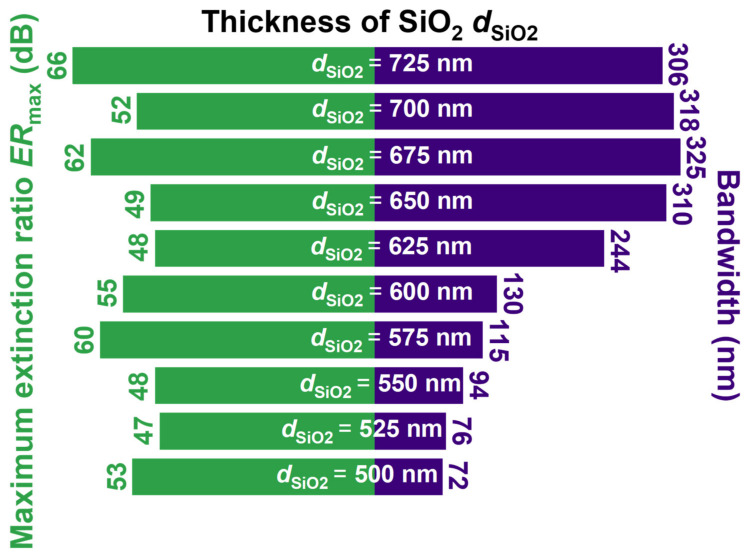
The bandwidth and the maximum extinction ratio *ER*_max_ within the corresponding wavelength region at the thickness of SiO_2_ *d*_SiO_2__ from 725 to 500 nm with the interval of 25 nm.

**Figure A5:**
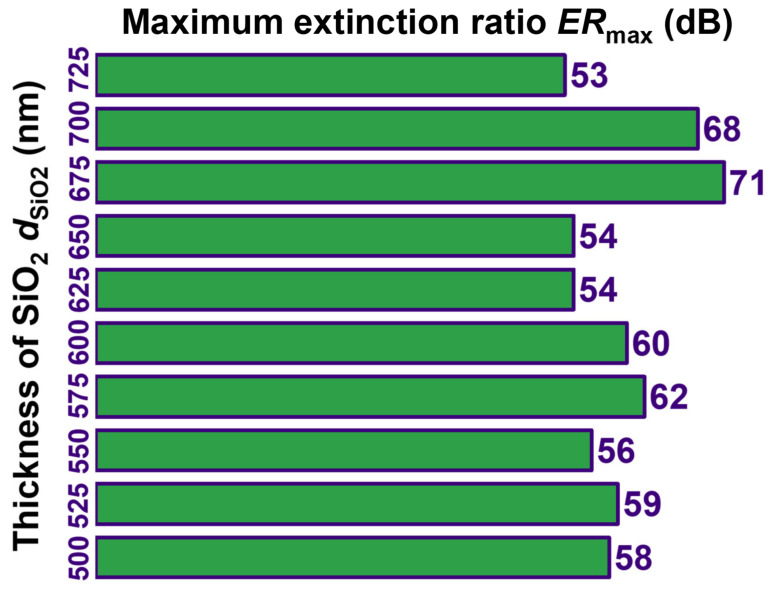
The maximum extinction ratio *ER*_max_ at the thickness of SiO_2_ *d*_SiO_2__ from 725 to 500 nm with the interval of 25 nm for separating p light at specific wavelength.

## Data Availability

The data presented in this study are available on request from the corresponding author.

## Appendix A. Mueller Matrix Spectroscopic Ellipsometry Analysis

Mueller matrix ellipsometric analysis is a model-based method, where the constructed and optimized optical model is empolyed to calculate Mueller matrix spectra to well match the measured ones. The optical model consists of not only the physical structure but also the dielectric tensor for each part. In ellipsometric analysis of the SnSe single crystal sample synthesized by chemical vapor transmission (CVT) method [8], its physical structure is treated as the anisotropic substrate, without considering roughness or overlay model (such as effective medium approximation, EMA [57]). To obtain the dielectric tensor of this anisotropic substrate, the Tauc–Lorentz oscillators with physical implication have been adopted along the *b*- and *c*-axis of SnSe, while the Cauchy model has been employed along the *a*-axis, in consideration of the van der Waals (vdW) interactions [42]. The dielectric functions along *b*- and *c*-axis can be expressed as the sum form of several Tauc–Lorentz oscillators [22,43]:

(A1) $$\varepsilon_{m}(E)=\sum_{q}^{Q}\varepsilon_{\mathrm{Tauc}-\mathrm{Lorentz}}^{q}(A_{q},\eta_{q},E_{0,q},E_{g,q};E),\;m\;=\;b\;\mathrm{and}\;c$$

In Equation (A1), *E* is the photon energy with the unit of eV, and *Q* is the total number of oscillators; *A_q_*, *η_q_*, *E*_0,*q*_, and *E*_g,*q*_ are, respectively, the *q*th oscillator’s amplitude, damping coefficient, peak transition energy, and bandgap energy. Specifically, the *q*th Tauc–Lorentz oscillator is composed of the real part *ε*_r,*q*_ and imaginary part *ε*_I,*q*_ in Equation (A2):

(A2) $$\varepsilon_{\mathrm{Tauc}-\mathrm{Lorentz}}^{q}(E)=\varepsilon_{r,q}(E)-i\varepsilon_{i,q}(E).$$

Equation (A3) is the specific expression of *ε*_i,*q*_ [22,43] while the *ε*_r,*q*_ can be converted from *ε*_i,*q*_ by Kramers–Kronig relation [22] of Equation (A4):

(A3) $$\varepsilon_{i,q}(E)=\left\{\begin{array}{l} \frac{A_{q}\eta_{q}E_{0,q}{\left(E-E_{g,q}\right)}^{2}}{{\left(E^{2}-E_{0,q}^{2}\right)}^{2}+\eta_{q}^{2}E^{2}}\cdot \frac{1}{E};\;\;E>E_{g,q}\\ 0.\;\;\;\;\;\;\;\;\;\;\;\;\;\;\;\;\;\;\;\;\;\;\;\;\;E\leq E_{g,q} \end{array}\right.$$

(A4) $$\varepsilon_{r,q}(E)=\varepsilon_{r,q}(\infty )+\frac{2}{\pi }P\int_{E_{g,q}}^{\infty }\frac{\xi \varepsilon_{i,q}(\xi )}{\xi^{2}-E^{2}}d\xi$$

The dielectric function along the *a*-axis is described by the Cauchy model [22,42], defined by the refractive index *n* and the extinction coefficient *k* in Equation (A5):

(A5) $$n(E)=A+BE^{2}+CE^{4};\;k\;=\;0$$

where *A*, *B*, and *C* represent the analytical parameters of the Cauchy model.

## Appendix B. Thickness-Dependent Bandgap of SnSe

We calculated the band structure of 2D SnSe from l layer to 5 layers (1 L–5 L) (Figure A1a–e) and bulk SnSe (Figure A1f) by first-principle, using the Perdew–Burke–Ernzerhof (PBE) functional and considering the spin–orbit coupling (SOC). It can be observed from Figure A1g that the valence band maximum (VBM) energy *E*_VBM_ remains almost unchanged and the conduction band minimum (CBM) energy *E*_CBM_ decline with the increase of the number of layers. Therefore, the bandgap energy *Eg* decreases with the increase of the number of layers, and the differences of *Eg* between 5 L SnSe and bulk SnSe is only 0.02 eV. The relation between *Eg* and number of layers share the same trend with the reported results [58].

## Appendix C. Optimization of the Thickness of SnSe and SiO_2_

At first, the relatively large interval thickness of SiO_2_ (250 nm) is selected to preliminarily figure out the variation of reflectance of p and s light and extinction ratio at the incident angle of 75° in Figure A2. As the thickness of SiO_2_ layer is 250 nm, the alternately bright and shaded stripes emerge in both reflectance of s and p light (Figure A1a,b, respectively) with the increase of SnSe’s thickness, due to the interference effect [59,60] in multilayer structure. The bright stripes becomes weaker and weaker when the SnSe’s thickness get thicker and thicker, which possibly originates from the stronger absorption of the thicker SnSe layer. In addition, by reason of both the extinction coefficient along the *b*- and *c*-axis of SnSe decreasing with the increase of near-infrared wavelength in Figure 2e, the absorption instead of interference in thicker SnSe layer is dominant in the shorter wavelength region of Figure A2a,b. Therefore, the stripes become shorter in the thicker SnSe layer and the shorter wavelength region in Figure A2a,b, respectively. More importantly, the optical anisotropy of SnSe brings the delayed reflectance of p or s light, which may lead to the high extinction ratio *ER* in Figure A2c. We select two representative thicknesses of SnSe layer (*d*_SnSe_ = 670 and 280 nm), corresponding to the maximum vaule of extinction ratio *ER*_max_ (above 50 dB) of Figure A2c to further illustrate the variation of reflectance of p and s light in Figure A2d. The wavelength corresponding to the s light’s maximum reflectance *R*_s,max_ (about 0.75) perfectly matches that of p light’s minimum reflectance *R*_p,min_ in both two kinds of thickness of SnSe layer. Therefore, the maximum extinction ratio results from delaying reflectance of p or s light to make their maximum and minimum correspond to the same wavelength. Besides, the p light’s minimum reflectance wavelength increases with the *d*_SnSe_, indicating modulating the *d*_SnSe_ is a potential and effective regualtion method.

The circumstance for SiO_2_’s thickness of 750 nm in Figure A2e–h is almost the same as that of SiO_2_’s thickness of 250 nm, and the major difference is the representative thicknesses of SnSe layer corresponding to the *ER*_max_, other than the increased interference within the same thickness of SnSe layer in Figure A2e,f. Excitingly, the *R*_p,min_ wavelength is from about 1400 nm to 1690 nm (marked area) at SnSe’s thickness of 450 nm, offering significant potential to realize the broadband modulation.

When the SiO_2_’s thickness is 1250 nm, the interference levels increase with the SiO_2_’s thickness, and there are two, even three times, of destructive interference in most the same thickness of SnSe layer in Figure A2i,j. Although the *R*_p,min_ wavelength is broadband at SnSe’s thickness of 390 nm in Figure A2l, its bandwidth (50 nm) is much smaller than that of SiO_2_ and SnSe’s thicknesses of 750 and 450 nm, respectively.

According to the analysis above, the interval of SiO_2_’s thickness is further shortened to 25 nm, and SiO_2_’s thickness is selected as 675, 700, and 725 nm to acquire longer bandwidth of *R*_p,min_ wavelength.

In Figure A3a, the SiO_2_’s thickness is 675 nm, and the two representative thicknesses of SnSe layer (*d*_SnSe_ = 400 and 315 nm), corresponding to the maximum alue of extinction ratio *ER*_max_, are selected to illustrate their reflectance *R*, exticntion ratio *ER*, and insertion loss *IL* at these two thicknesses of SnSe layer in Figure A3b,c, respectively. In Figure A3b, the *R*_s_ is above 0.6 and the *IL* is consequently below 2 dB in the whole concerned wavelength region. The p light can be separated over broadband since *R*_p_ low than 0.003 within the wavelength region from 1262 to 1586 nm. Therefore, the *ER* maintains a high level of about 23 dB at such wavelength region with its maximum *ER*_max_ over 60 dB. When the thickness of SnSe layer becomes thinner (*d*_SnSe_ = 315 nm), the *R*_s_ gets much higher, and the *IL* is lower than 1.5 dB from the wavelength of 1000 nm to that of 1200 nm as a result in Figure A3c. However, the *R*_p_ reaches its minimum at the wavelength of about 1100 nm accompanied by the *ER*_max_ over 70 dB, which means p light can be separated at a specific wavelength.

The circumstances for the SiO_2_’s thickness of 700 and 725 nm are similar to that of SiO_2_’s thickness of 675 nm, and Table A1 and Table A2 summarize their differences.

**Table A1:** Performances of separating p light over broadband with thicker SnSe’s thickness (*d*_SnSe_ = 400, 430 and 455 nm) respectively at the SiO_2_’s thickness of 675, 700, and 725 nm.

Thickness of SiO_2_ [nm]	Thickness of SnSe [nm]	Wavelength Region ^1^ [nm]	Bandwidth [nm]	Maximum Extinction Ratio *ER*_max_ [dB]	Average Insertion Loss *IL*_ave_ [dB]
675	400	1262−1586	324	62	1.7
700	430	1325−1642	317	52	1.7
725	455	1383−1688	305	66	1.8

^1^ Corresponding to wavelength region of *R*_p_ < 0.003.

**Table A2:** Performances of separating p light at specific wavelength with thinner SnSe’s thickness (*d*_SnSe_ = 315, 290 and 280 nm) respectively at the SiO_2_’s thickness of 675, 700, and 725 nm.

Thickness of SiO_2_ [nm]	Thickness of SnSe [nm]	Wavelength ^1^ [nm]	Maximum Extinction Ratio *ER*_max_ [dB]	Insertion Loss *IL* [dB]
675	315	1100	71	1.1
700	290	1043	68	1.2
725	280	1027	53	1.1

^1^ Corresponding to the wavelength of *ER*_max_.

In Table A1 and Table A2, both the average insertion loss *IL*_ave_ and the *IL* at the wavelength of *ER*_max_ are lower than 2 dB, suggesting the high s light’s reflectance *R*_s_ (above 0.63). Although the *ER*_max_ for the SiO_2_’s thickness of 675 nm is slightly lower than that for the SiO_2_’s thickness of 725 nm, its bandwidth of *R*_p_ < 0.003 is longest among three kinds of thickness of SiO_2_ in Table A1. Additionally, its *ER*_max_ in Table A2 is also the highest. Hence, compared with the other two kinds of thicknesses of SiO_2_ (*d*_SiO_2__ = 700 and 725 nm), the SnSe-SiO_2_-Si multilayer structure with the SiO_2_’s thickness of 675 nm displays relatively highest performances.

We also calculate the bandwidth and the maximum extinction ratio *ER*_max_ within it at the thickness of SiO_2_ *d*_SiO_2__, from 725 to 500 nm, with the interval of 25 nm in Figure A4 to find out the optimal *d*_SiO_2__. With the reduction of SiO_2_’s thickness, the bandwidth at first increases to 325 nm at the SiO_2_’s thickness of 675 nm, and then gradually decreases until the SiO_2_’s thickness of 500 nm. Therefore, the bandwidth corresponding to the SiO_2_’s thickness of 675 nm is the longest, whose *ER*_max_ is 62 dB, and is the second highest among these thicknesses of SiO_2_. As shown in Table A1, the insertion losses of three kinds of SiO_2_’s thickness (*d*_SiO_2__ = 675, 700, and 725 nm) are relatively small and almost the same; hence, the insertion losses may not significantly influence the performances of separating p light over broadband and are not taken into consideration for the thinner thickness of SiO_2_.

For separating p light at specific wavelength, the *ER*_max_ shares the same trend as the bandwidth in Figure A5, and the *ER*_max_ at the SiO_2_’s thickness of 675 nm is the highest as a result.

According to the analysis above, the SiO_2_’s thickness is selected as 675 nm, and the corresponding SnSe’s thickness is 400 and 315 nm for separating p light over broadband and at specific wavelength respectively.
